# Physical activity and neuroinflammation: a bibliometric analysis of research progress and future perspectives

**DOI:** 10.3389/fnagi.2025.1602724

**Published:** 2025-08-04

**Authors:** Yeting Zhang, Huangyan Li, Jiangxi Yang, Huan Ma

**Affiliations:** ^1^Department of Sports, Civil Aviation Flight University of China, Guanghan, China; ^2^College of Education Science, Sichuan Normal University, Chengdu, China

**Keywords:** neuroinflammation, neurodegenerative disease, physical activity, exercise, bibliometric analysis

## Abstract

**Background:**

Neuroinflammation is a common pathological feature of neurodegenerative and psychiatric diseases and is closely related to the dysfunction of the nervous system. In recent years, an increasing number of studies have shown that physical activity (PA) has a significant regulatory effect on neuroinflammation. However, a comprehensive analysis of research in this field is currently lacking, including the evolution of knowledge structures, interdisciplinary trends, and dynamic shifts in research hotspots.

**Methods:**

This study retrieved relevant literature from the Web of Science Core Collection database for the period from 2004 to 2025. The search strategy was TS = ((“physical activit*” OR exercis* OR “exercise training”) AND (“neuroinflammat*” OR “neuro inflammatory” OR “neuro-inflammatory”)), with the document type limited to Articles and Reviews. After screening, a total of 661 eligible articles were included for bibliometric analysis. The analysis tools used were the Bibliometrix R package and VOSviewer, which were employed to visualize the results of the literature analysis.

**Results:**

From 2004 to 2025, the number of publications in this field showed a yearly increasing trend, with an annual growth rate of 15.05%. China and the United States were the main contributing countries, publishing 122 and 111 articles, respectively. In terms of journals, the *International Journal of Molecular Sciences* ranked first with 36 articles and a total of 799 citations. Among the institutions, Karolinska Institute led the way in terms of citation counts, amassing a total of 391 citations. Regarding author keywords, “Alzheimer’s disease,” “microglia,” and “older adults” were the three most frequently occurring keywords. Research hotspots have gradually shifted from the early focus on hippocampal function and neuroinflammation mechanisms to current directions such as neurodegenerative diseases, microglial regulation, and the gut–brain axis.

**Conclusion:**

This study systematically reviewed the research progress in the field of PA and neuroinflammation from 2004 to 2025 using bibliometric methods and revealed the research hotspots, trends, and thematic evolution in this field. It provides a systematic scientific basis for scholars to understand the field, optimize research directions, and develop intervention strategies.

## 1 Introduction

Neuroinflammation (NI) is a common pathological feature of neurodegenerative and psychiatric diseases and is closely related to the dysfunction of the nervous system. It provides a systematic scientific basis for scholars to understand the field, optimize research directions, and develop intervention strategies (Lee et al., 2023). In the central nervous system (CNS), microglia, as innate immune cells, play a key role by being activated in response to neuroinflammatory stimuli (Isik et al., 2023). Meanwhile, astrocytes are also involved, with their reactions being either pro-inflammatory or anti-inflammatory, depending on the type of stimulus in the inflammatory environment (Kwon and Koh, 2020). Microglia can respond to and propagate peripheral inflammatory signals, triggering low-grade inflammatory responses within the brain, which in turn lead to changes in neuronal activity and ultimately result in physiological and behavioral impairments (Woodburn et al., 2021). This series of events is accompanied by the activation, synthesis, and release of pro-inflammatory cytokines and growth factors, which drive the progression of various neurodegenerative diseases (NDs), thereby promoting the progression of various NDs, including Alzheimer’s disease (AD), Parkinson’s disease (PD), and multiple sclerosis (MS) (Giri et al., 2024; Xu et al., 2024).

In recent years, an increasing number of studies have demonstrated that physical activity (PA) exerts a significant regulatory effect on neuroinflammation. Exercise, as a simple and effective intervention, can create an anti-inflammatory environment in peripheral organs and the brain, thereby positively influencing diseases associated with neuroinflammation. For instance, exercise has been shown to alleviate cellular damage and cognitive impairment in AD by modulating neuroinflammation (Pahlavani, 2023). The underlying mechanisms primarily involve the inhibition of microglial activation and the induction of an anti-inflammatory state, which subsequently reduces pro-inflammatory cytokine levels while increasing the expression of anti-inflammatory cytokine receptors (Mee-Inta et al., 2019). Moreover, both animal models and human studies have indicated that PA can effectively regulate inflammatory responses and ameliorate symptoms of neuroinflammatory-related diseases.

However, despite existing studies that have elucidated the relationship between PA and NI, a comprehensive analysis of the research field of PA and NI is still lacking, including the evolution of knowledge structures, interdisciplinary trends, and the dynamic shifts in research hotspots. Bibliometrics, through the quantitative analysis of the spatiotemporal distribution of scientific literature, collaborative networks, and thematic evolution, offers a unique perspective for revealing the developmental patterns of academic disciplines. Although some studies have explored the effects of PA in neuroscience, a systematic bibliometric analysis targeting the field of PA and NI remains a gap in the literature. Therefore, the present study aims to systematically review the research progress in the field of PA and NI from 2004 to 2025 using bibliometric methods, analyze research hotspots, trends, and thematic evolution, and provide direction and reference for future studies.

## 2 Materials and methods

### 2.1 Data sources and search strategy

**FIGURE 1:**
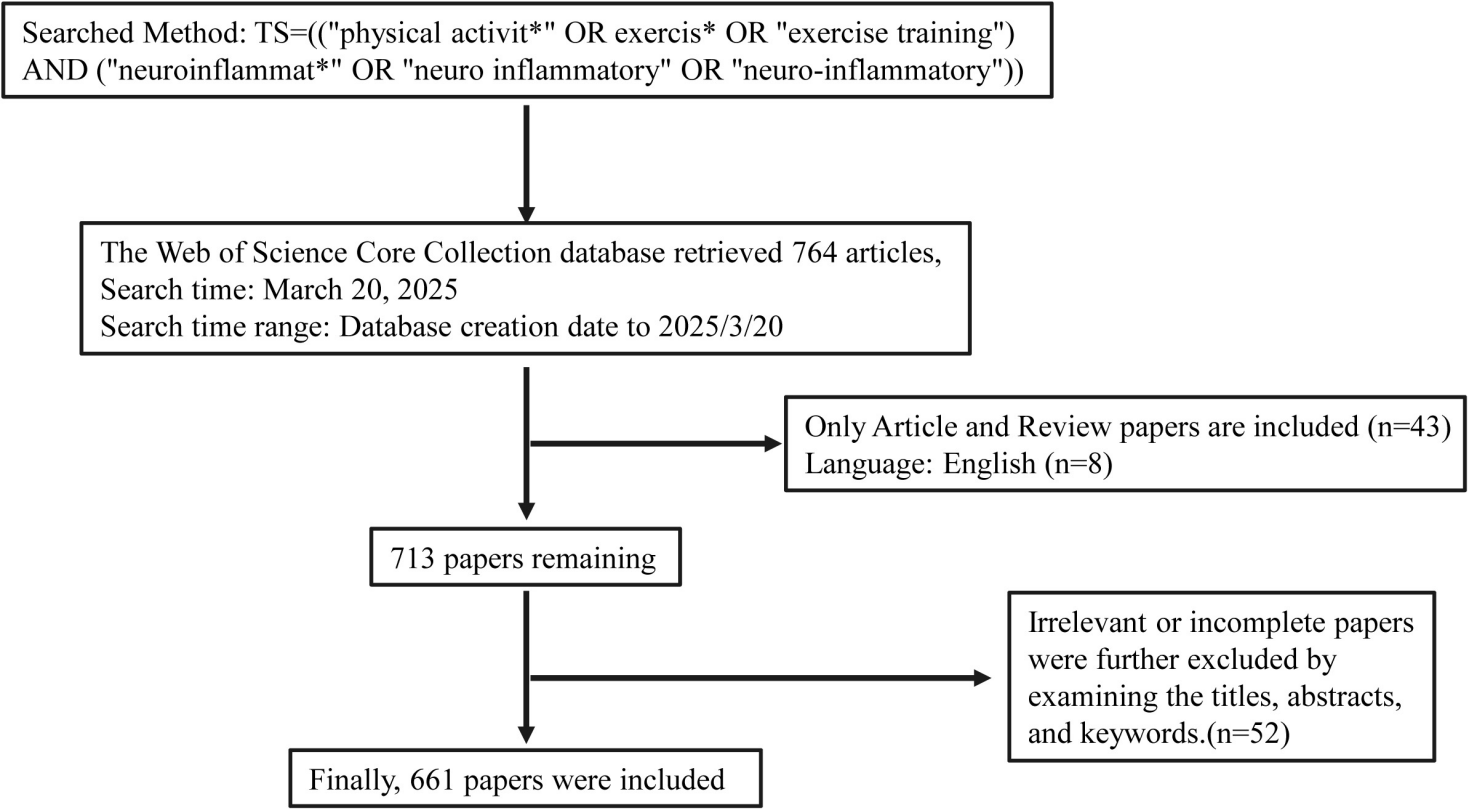
Flow diagram for the screening.

This study conducted a literature search using the Web of Science Core Collection (WoSCC) database. The search strategy was formulated as follows: TS = ((“physical activit*” OR exercis* OR “exercise training”) AND (“neuroinflammat*” OR “neuro inflammatory” OR “neuro-inflammatory”)), with the document type restricted to Article or Review. The search spanned from the inception of the database to 20 March 2025, covering databases including SCI-EXPANDED, SSCI, A&HCI, CPCI-S, CPCI-SSH, BKCI-S, BKCI-SSH, ESCI, CCR-EXPANDED, and IC, among others. After multiple reviews and manual screening of the search results, this search strategy was finalized. A total of 764 articles relevant to the research field were retrieved. During the data processing phase, two researchers independently conducted the literature search, download, and verification to ensure accuracy. In the screening process, non-English articles, duplicate publications, articles with incomplete bibliographic information, articles not relevant to the research topic, as well as non-scholarly content such as editorials, news, letters, and conference abstracts, were excluded. Ultimately, 661 articles that met the research criteria were included. The literature screening process is detailed in Figure 1.

### 2.2 Data analysis

In this study, we employed bibliometric tools such as the Bibliometrix R package and VOSviewer to visualize our literature analysis results (Aria and Cuccurullo, 2018). To better present our research findings, we carefully selected software suitable for various aspects of our study. Specifically, we utilized R (version 4.1.2), the R-bibliometrix package (version 3.0.3),^1^ and VOSviewer software (version 1.6.18) to visualize the selected literature (Hu et al., 2024). With these tools, we conducted in-depth analyses of publications, countries, institutions, journals, and keywords. Using the R-bibliometrix package, we first performed frequency statistics on journals, institutions, and countries, including the number of publications and citation counts. For each category, we listed the top 20 frequencies. Secondly, we conducted keyword trend analysis, during which we created two auxiliary files (in txt format): a synonym file for merging synonyms (e.g., “older adults,” “ageing,” “aging,” “aged,” “older-adults,” “elderly,” “elderly persons,” “elderly-people,” “older-people,” and “older people” were merged into “older adults”) and a removal file for eliminating duplicate or unnecessary keywords (such as search terms “physical activity,” “exercise,” “exercise training,” “neuroinflammation,” etc.). The analysis focused on the dynamic evolution of research topics from 2004 to 2025. Specifically, by meticulously analyzing keyword frequencies, we captured their temporal changes and drew thematic maps for two specific periods to intuitively illustrate the development and transformation trends of research topics. It is worth emphasizing that our study adopted an integrated platform-based comprehensive analysis method, which ensured high methodological consistency and good reproducibility throughout the research process (Hu et al., 2024). For the parameter settings of the remaining software, we used the default values. When running R-bibliometrix, we employed Biblioshiny, a user-friendly web interface for R-bibliometrix, which facilitates data visualization and the display of social network diagrams. Additionally, we used VOSviewer software (version 1.6.16, developed by Nees Jan van Eck and Ludo Waltman from Leiden University),^2^ to generate co-occurrence networks of author collaboration, countries, institutions, and keywords. In these networks, the size of the nodes represents the frequency of occurrence or citation counts, and the thickness of the connecting lines reflects the strength of collaboration. During the keyword clustering analysis, we utilized Modularity Optimization in the VOSviewer software. This algorithm determines the optimal clustering solution by optimizing the modularity score, thereby ensuring the robustness and objectivity of the clustering outcomes. We set the minimum frequency of occurrence for institutions, journals, and keywords to 10. For countries, the minimum frequency was set to 5, and for article citation counts, the minimum frequency was set to 100.

## 3 Results

### 3.1 Overview of publications and time trends

**FIGURE 2:**
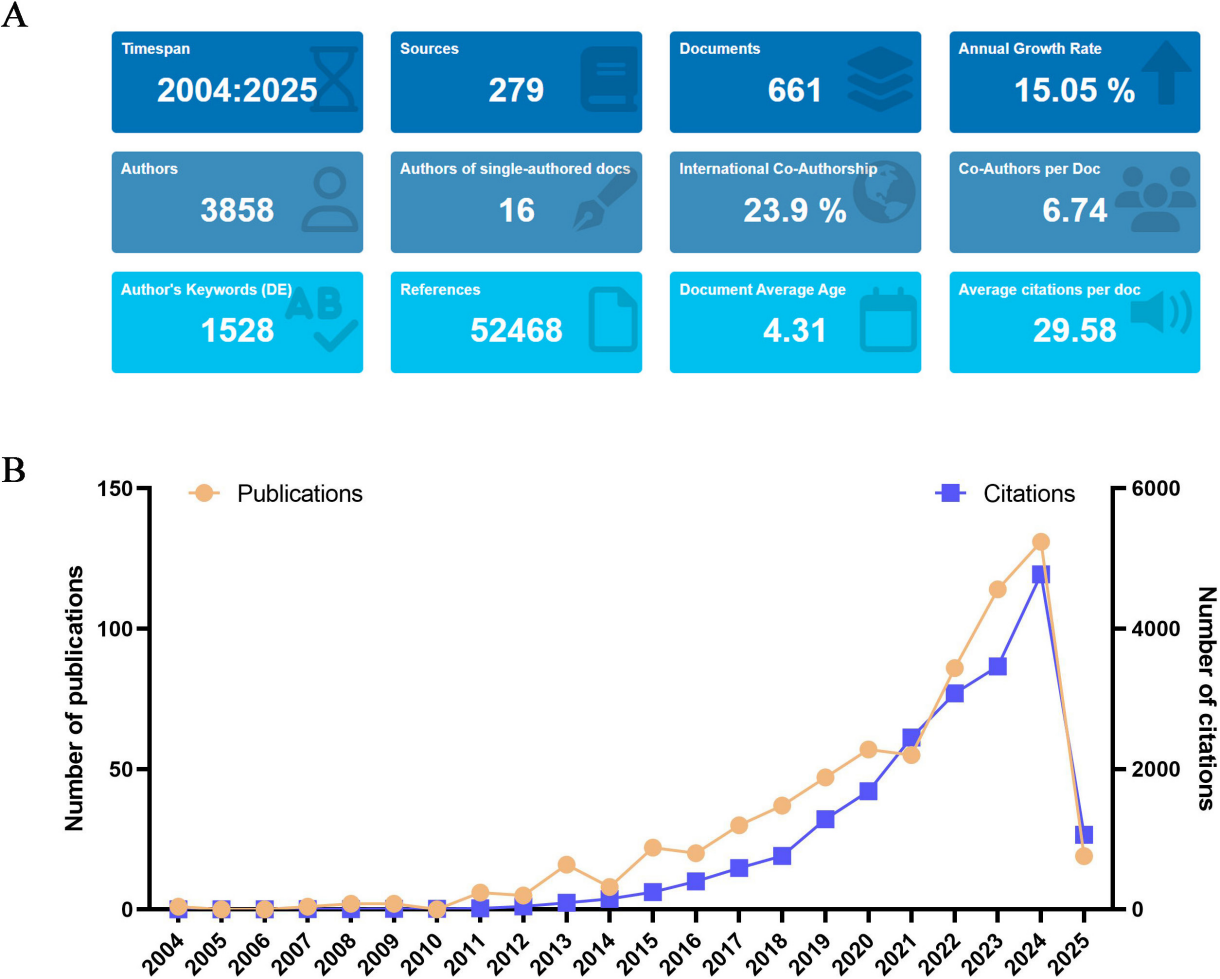
Descriptive analysis of the retrieved literature. **(A)** Main information of 661 included articles related to PA and NI. **(B)** Trends in the number of publications and total citations.

A total of 764 articles were retrieved from the WoSCC database. After screening and evaluating the eligibility based on the criteria, 661 articles related to PA and NI were finally included for bibliometric analysis (see Figure 2A). These articles were authored by 3,858 researchers from 200 institutions across 68 countries, published in 279 journals, and cited 52,468 references from 5,220 journals. From 2004 to 2025, the annual number of publications increased slightly, with a yearly growth rate of 15.05%. As shown in Figure 2B, a noticeable peak in publications occurred in 2013 (*n* = 16). After 2021, the number of publications increased sharply, with the highest number of publications in 2024 (*n* = 131), and this trend is expected to continue.

### 3.2 Analysis of countries

**FIGURE 3:**
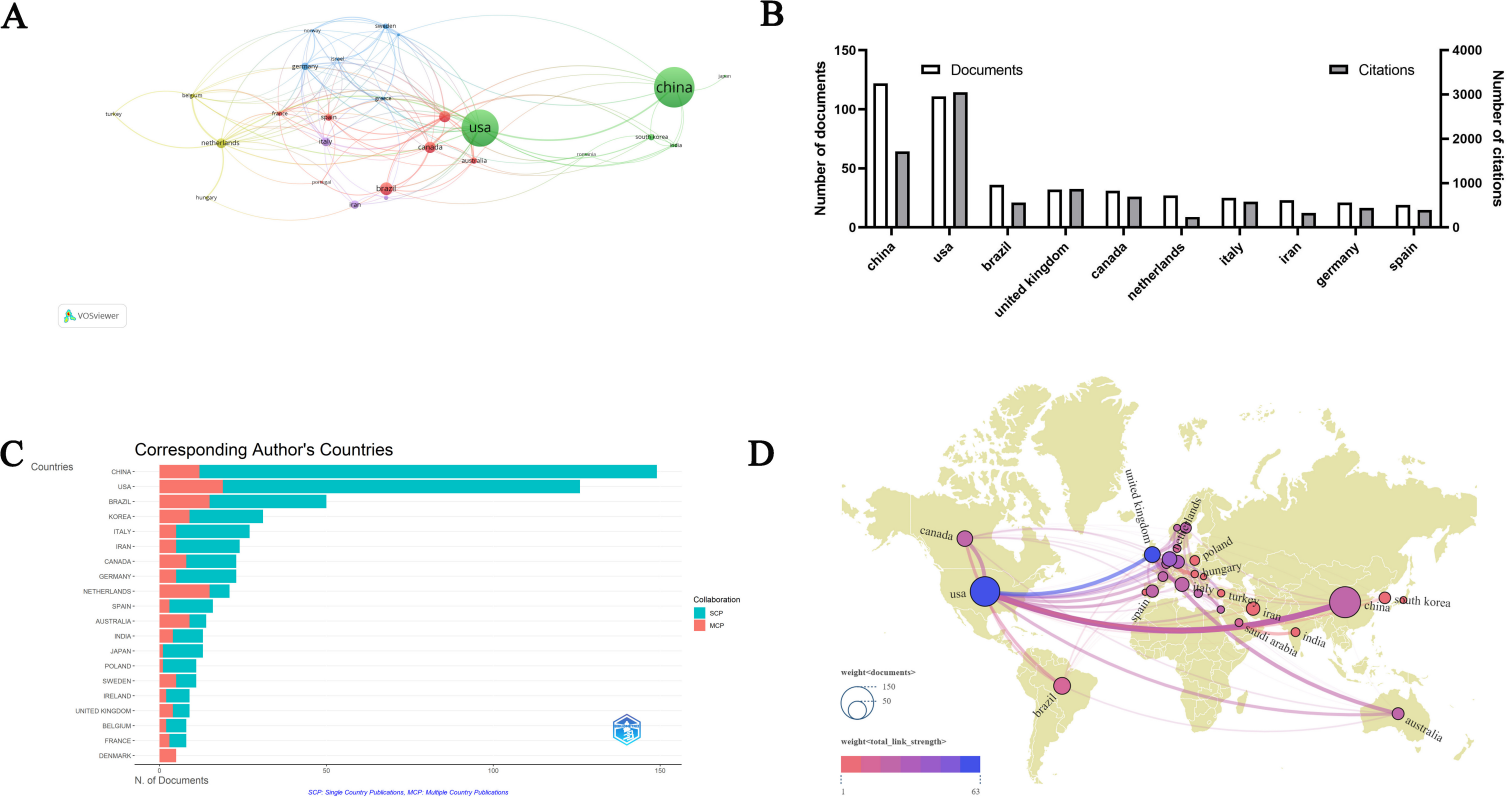
Analysis of countries. **(A)** Network visualization of countries collaborations. **(B)** The 10 leading countries ranked by publication volume. **(C)** The location of published articles based on the corresponding author’s country. **(D)** Co-authorship analysis illustrated in the geographical map.

Approximately 68 countries have contributed to research on PA and NI. Among them, China and the United States have published over 100 articles each, significantly surpassing other countries. A total of 21 countries have published more than 10 articles, with 9 countries publishing over 30 studies. However, 20 countries have published only a single article. As shown in Figures 3A, B, China has the highest number of publications in this field, with 122 articles (accounting for 18.46% of the total), followed by the United States with 111 articles (16.79% of the total). Other notable countries include Brazil, the United Kingdom, Canada, the Netherlands, Italy, Iran, Germany, and Spain. The United States also has the highest total citation count (*n* = 3,052) and the highest average citation per article (*n* = 27.49). Although China has the highest number of publications, its citation count still lags behind that of the United States (see Figure 3B). China produced the highest number of single-country publications (137 articles), whereas the United States led in multi-country collaborations (19 articles). The Netherlands has the highest proportion of multi-country publications, with 6 single-country articles and 15 multi-country articles (see Figure 3C). Co-authorship analysis among these countries identified the United States (TLS = 65), the United Kingdom (TLS = 49), and the Netherlands (TLS = 42) as the top three countries in terms of collaboration levels. Among them, the United States has the most robust collaborative network with European countries and regions (see Figure 3D).

### 3.3 Analysis of institutions

**FIGURE 4:**
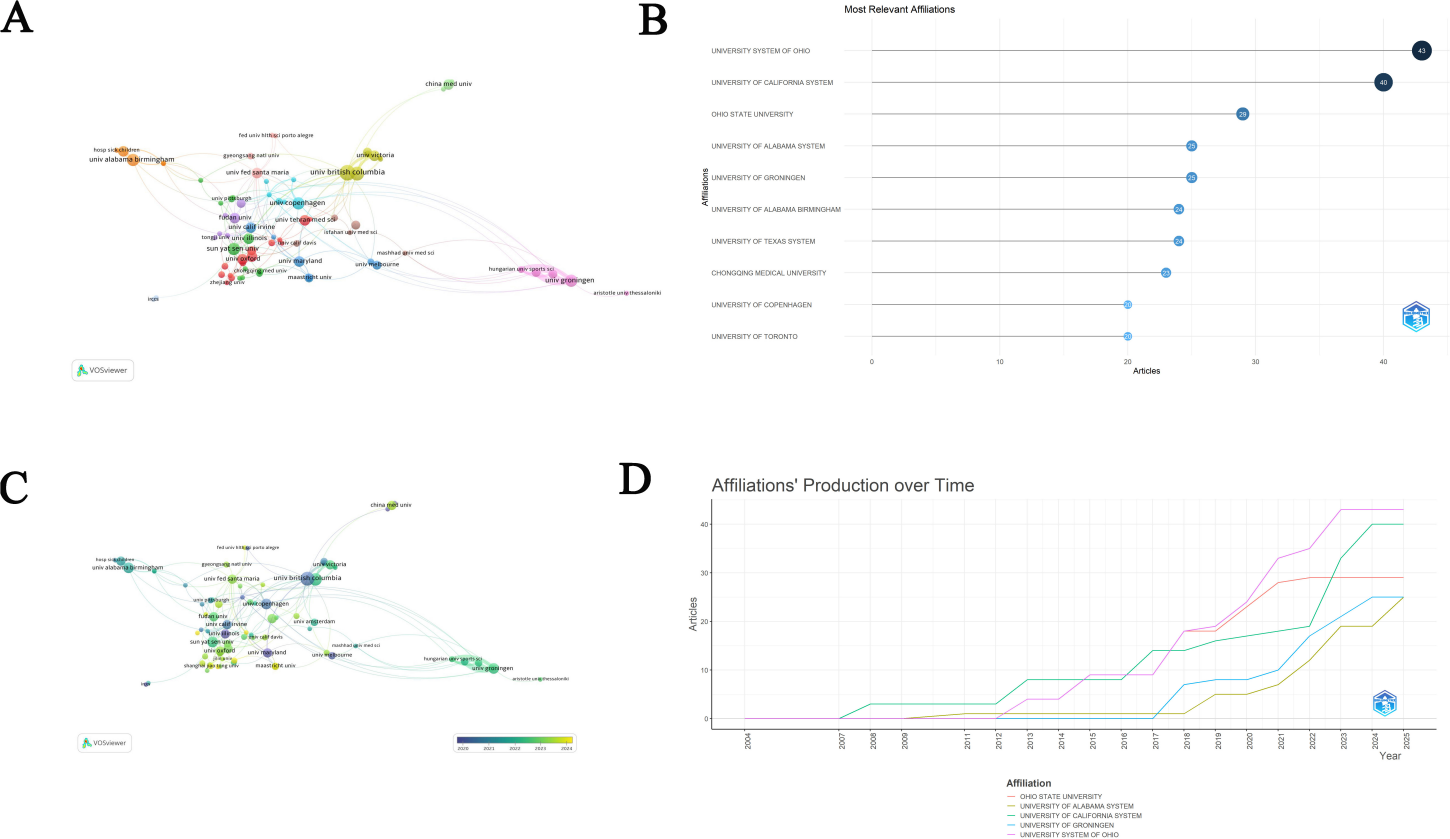
Analysis of institutions. **(A)** Network visualization of institutional collaborations. **(B)** The top 10 most contributing institutions. **(C)** The overlay visualization of bibliographic coupling between institutions. **(D)**Annual publication trends of the top five institutions.

A total of 200 institutions have published articles on PA and NI. Among them, 26 institutions contributed more than five papers. The collaborations of these 26 institutions were visualized through co-authorship analysis, creating network visualizations of institutional collaborations and overlay visualizations of bibliographic coupling between institutions (Figures 4A, C). Utilizing VOSviewer, we conducted a citation analysis of the papers published by each institution. The top five institutions in terms of citation counts are Karolinska Institute (391 citations), University of Copenhagen (387 citations), Sun Yat-sen University (277 citations), University of Illinois (254 citations), and University of Maryland (235 citations). In addition to citation analysis, we also employed the number of published papers as an indicator to provide a more comprehensive assessment of institutional contributions. The top 10 institutions ranked by publication volume included the Ohio University, which had the highest number of publications at 43 articles, followed by the University of California (*n* = 40) and Ohio State University (*n* = 29) (Figure 4B). VOSviewer conducted a bibliographic coupling analysis of institutions that published more than five papers, revealing that institutions such as University of Illinois, University of Maryland, and University of British Columbia had higher publication volumes in the early stages. In contrast, in recent years, institutions like University of Oxford, Wuhan University, and China Medical University have shown increased publication output (Figure 4C). The top five institutions all experienced a significant increase in the number of articles published after 2017, which may reflect a substantial improvement in research productivity during this period (Figure 4D). Considering the citation data, institutions such as Karolinska Institute, University of Copenhagen, and Sun Yat-sen University have made significant contributions to the field.

### 3.4 Analysis of journals

**FIGURE 5:**
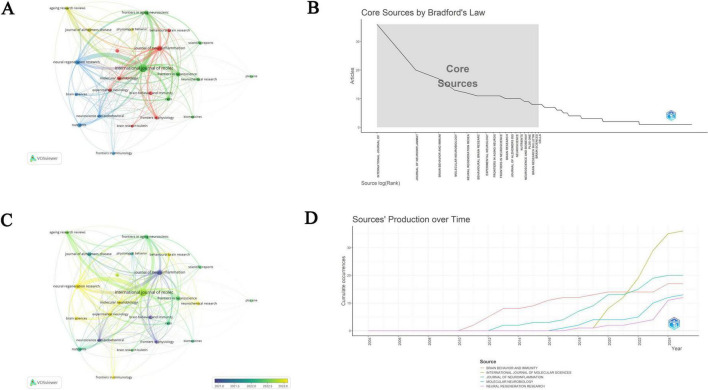
Analysis of journals. **(A)** Network visualization illustrating academic journals collaborations. **(B)** Core journals by Bradford’s law. **(C)** The overlay visualization of bibliographic coupling between academic journals. **(D)** Annual publication trends of the top five journals.

As shown in Table 1, the *International Journal of Molecular Sciences* demonstrated the highest academic impact in this field, with the highest h-index (15), which is strongly correlated with its leading position in publication output (36 articles) and total citations (799). Following closely are *Brain Behavior and Immunity and Journal of Neuroinflammation*, both with an h-index of 14, having published 17 and 20 articles respectively, and with total citations of 1,131 and 1,241 respectively. This pattern is consistent across the entire journal domain, indicating a strong positive correlation between a journal’s h-index and its publication output and citation counts. A total of 279 journals published articles on PA and NI, among which 24 journals contributed more than 5 articles. Through co-authorship analysis, these 24 journals were visualized to create a network visualization of institutional collaborations and an overlay visualization of bibliographic coupling between institutions (Figures 5A, C). The bibliographic coupling analysis revealed that journals with a higher number of publications in the early stages included *Journal of Neuroinflammation*, *Frontiers in Physiology*, and *Neuroscience and Biobehavioral Reviews*. In contrast, in recent years, journals such as *Neural Regeneration Research*, *Molecular Neurobiology*, and *Behavioural Brain Research* have shown a higher number of publications (Figure 5C). Figure 5B shows that the core area consists of 18 high-quality journals, accounting for 6.45% of the total number of journals, which collectively published 224 articles according to Bradford’s law. Notable journals in the core area include *International Journal of Molecular Sciences*, *Journal of Neuroinflammation*, and *Brain Behavior and Immunity*. The top five journals all showed varying degrees of growth after 2010, with the most significant increase observed in *International Journal of Molecular Sciences* in recent years. This may reflect a substantial improvement in the research output of these journals in the relevant field (Figure 5D).

**TABLE 1:** Top 10 most influential journals.

Rank	Journal	h_index	TC	NP
1	International Journal of Molecular Sciences	15	799	36
2	Brain Behavior and Immunity	14	1,131	17
3	Journal of Neuroinflammation	14	1,241	20
4	Neuroscience	9	325	10
5	Brain Research	8	344	10
6	Frontiers in Aging Neuroscience	8	260	11
7	Behavioural Brain Research	7	309	11
8	Cells	7	246	8
9	Frontiers in Neuroscience	7	199	11
10	Molecular Neurobiology	7	713	13

TC, total citations; NP, number of publications.

### 3.5 Author keywords frequency and trend topics

**FIGURE 6:**
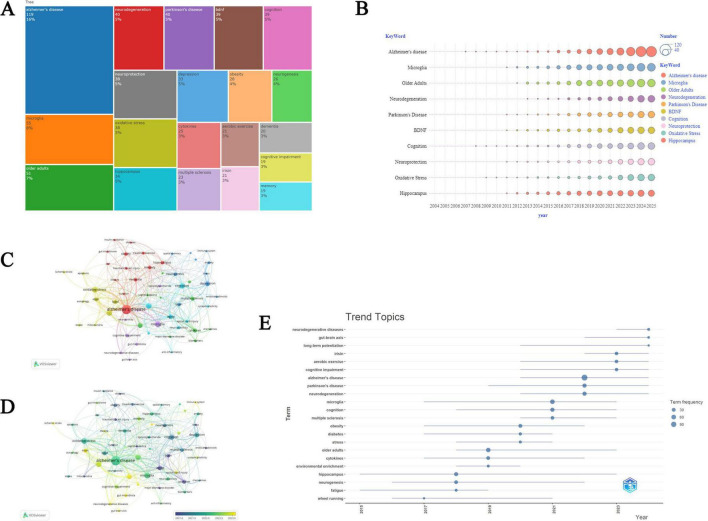
Analysis of keywords. **(A)** Tree map of the most frequent author keywords. **(B)** Annual publication trends of the top 10 keywords. **(C)** Network visualization illustrating of keywords. **(D)** The overlay visualization of keywords. **(E)** Trend topics in the research domain of PA and NI.

In the research domain of PA and NI, we identified the top 20 author keywords (Figure 6A). The top three author keywords in the dataset are “Alzheimer’s disease,” “microglia,” and “older adults,” with occurrence frequencies of 119, 55, and 51 times, respectively, accounting for 16%, 8%, and 7% of all keywords. Additionally, “neurodegeneration” and “Parkinson’s disease” each appeared 40 times, representing approximately 5% of the total occurrences. Among the top 20 author keywords identified, “BDNF,” “cognition,” “neuroprotection,” and “oxidative stress” are also frequently occurring terms, with 39, 39, 38, and 38 occurrences, respectively, each accounting for about 5% of the total terms. Figure 6B presents a bubble chart illustrating the temporal trends of the most frequent keywords from 2004 to 2025, showing the frequency and trend of these keywords in research publications. There are a total of 1,528 unique keywords in articles related to PA and NI, with 213 keywords occurring more than five times. These 213 keywords were visualized through keyword analysis, creating a network visualization and an overlay visualization of bibliographic coupling between keywords (Figures 6C, D). Trend topic analysis revealed the temporal evolution patterns in the research domain (Figure 6E). From 2015 to 2024, research topics exhibited significant dynamic changes. Early research topics such as “fatigue” and “hippocampus” emerged around 2015, with “hippocampus” having a particularly long time span of occurrence, lasting until around 2023. During the period from 2019 to 2021, several core research directions emerged, including “Alzheimer’s disease,” “Parkinson’s disease,” and “neurodegeneration.” The most recent research hotspots (2021–2023) have primarily focused on “long-term potentiation,” “gut–brain axis,” and “neurodegenerative diseases.” Notably, “Alzheimer’s disease” showed a high frequency of occurrence (90 times) and sustained attention throughout the research period. This pattern of topic evolution reflects the in-depth investigation of brain health and neurological diseases, as well as the urgent need to improve quality of life and address the challenges of an aging society.

**FIGURE 7:**
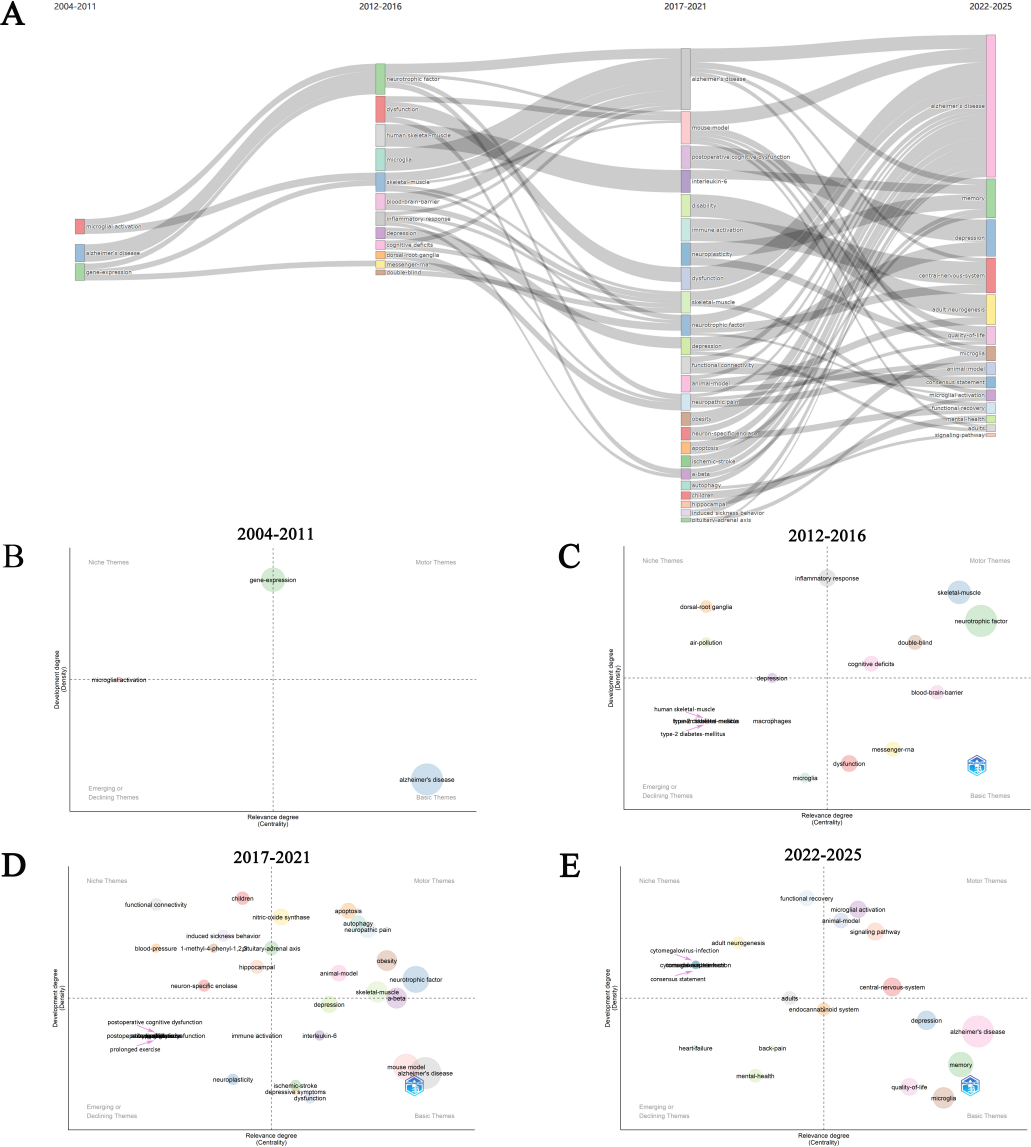
The evolution trend of keywords. **(A)** The changes and internal connections of keywords in different time periods. Thematic evolution within PA and NI in the periods 2004–2011 **(B)**, 2012–2016 **(C)**, 2017–2021 **(D)**, and 2022–2025 **(E)**.

Figure 7A illustrates the evolution and interrelationships of research topics across different time periods from 2004 to 2025. In the figure, nodes represent keywords, with the size of the nodes potentially correlating to the frequency of keyword occurrence. The lines connecting the nodes indicate co-occurrence relationships between keywords in the literature, and the thickness of these lines may reflect the strength of co-occurrence. The study period was divided into four intervals (2004–2011, 2012–2016, 2017–2021, and 2022–2025). This segmentation was based on a systematic analysis of the evolution of research topics (the specific characteristics of topic evolution are described in the following paragraph). Additionally, by analyzing the number of publications and citations each year, we identified two distinct inflection points in 2012 and 2017, which further support our temporal segmentation. Regrettably, there is currently no quantitative or objective method available to determine these time periods. This approach to segmentation may somewhat affect the precise interpretation of the evolutionary trends of research topics. However, considering the distribution characteristics of the number of publications and the phased features of the development of research topics, we believe that this segmentation method can, to some extent, reasonably reflect the evolutionary process of research topics.

Early studies (2004–2011) were relatively scattered, covering topics such as “microglial activation,” “Alzheimer’s disease,” and “gene expression.” During the period of 2012–2016, research topics began to diversify and evolve, with the emergence of new themes such as “microglia,” “dysfunction,” and “blood-brain barrier,” indicating a shift in focus toward specific biological mechanisms and disease models. In the 2017–2021 timeframe, the number of research topics further increased. The primary research trends during this period were concentrated on NDs (especially AD), microglia, neurodegeneration, cognitive function, oxidative stress, depression, immune activation, and neuroplasticity. These areas of research were highly active and interconnected. Over time, from 2022 to 2025, research topics became more focused, particularly on “Alzheimer’s disease” and “neurodegeneration.” There was a sustained and in-depth focus on NDs, cognitive function, microglia, depression, oxidative stress, and inflammation. These trends suggest that scientists are actively exploring the pathogenic mechanisms of these diseases and seeking effective treatments to address the challenges posed by global aging. Additionally, the widespread distribution and interconnections of keywords in the figure, such as “neurotrophic factor,” “dysfunction,” and “inflammatory response,” indicate that research in this field is increasingly interdisciplinary, involving multiple domains including neuroscience, immunology, and molecular biology.

Figures 7B–E illustrates the evolutionary trends of research themes in the field of PA and NI across different time periods. The thematic maps generated through keyword clustering provide a strategic overview of the research dynamics in this field. The thematic maps for each time period are divided into four quadrants. In the thematic maps, the horizontal axis represents centrality, which is the betweenness centrality of the theme within the co-occurrence network. Betweenness centrality measures the extent to which a node serves as a “bridge” among other nodes. A higher value indicates that the theme plays a more critical role in connecting different themes and is more relevant to the field. The vertical axis represents density, which is the internal connectivity strength of the theme. Specifically, it is the ratio of the sum of weights of co-occurrence edges within the theme to the maximum possible sum of weights. A higher density value indicates tighter internal connections within the theme and a higher level of development of the theme within the field (Dong et al., 2023).

In Figure 7B, during the period of 2004–2011, “gene-expression” is located in the quadrant of high development level and high relevance, indicating its significant research activity and centrality as a motor theme in this field. Meanwhile, “microglial activation n” and “Alzheimer’s disease” are respectively located in the quadrants of niche themes and basic themes, demonstrating their high development level in specific research directions and fundamental role within the entire field. Figure 7C (during 2012–2016) encompasses a broader range of research themes. “Skeletal-muscle” and “neurotrophic factor” serve as driving themes, highlighting their core position and significant development in this research area. In contrast, “dorsal-root ganglia” and “macrophages” are located in the quadrants of niche themes and emerging or declining themes, respectively, indicating their importance within specific research domains and potential developmental trends. Figure 7D (during 2017–2021) further refines the research themes. “Neurotrophic Factor,” “obesity,” “animal model,” and “Skeletal-Muscle” play central roles in this research field. Meanwhile, “neuroplasticity” and “immune activation,” as emerging or declining themes, may suggest that research in these areas is undergoing a transition, possibly experiencing either an upsurge or decline. “Mouse model” and “Alzheimer’s disease” are identified as basic themes, emphasizing their sustained importance and extensive relevance in this research area. Lastly, Figure 7E (during 2022–2025) demonstrates the active development of themes such as the CNS and microglial activation, while AD remains a foundational theme within this domain. Overall, these thematic maps, by illustrating the distribution and evolution of research themes across different time periods, provide a significant reference for understanding the research trends and future development directions in the field of PA and NI.

## 4 Discussion

The interplay between PA and NI has emerged as a critical research focus. Our study found that from 2004 to 2025, there was a significant increase in the annual number of publications and citation counts related to this field. Through bibliometric analysis, several key findings were identified, including analyses of PA and NI related literature based on citation counts, journals, institutions, countries/regions, author keyword frequencies, trending topics, and thematic evolution. In addition, trending topics related to the field of PA and NI were identified, and the thematic evolution within this research domain from 2004 to 2025 was delineated. Notably, we also conducted an analysis of the most influential articles and most influential authors, with detailed information provided in the Supplementary material.

Trend topic analysis can reflect the iterative nature of research hotspots in the field of PA and NI. Notably, influential themes began to emerge around 2015 and can be categorized into four distinct phases over time. Initially, research primarily focused on the impact of PA on neuroinflammation within the hippocampus, indicating that the hippocampus is a crucial brain region in this field of study (Fonken et al., 2016; Kang et al., 2016). The subsequent phase involved the effects of PA on inflammatory markers, specifically cytokines such as interleukin (IL)-6 and IL-15. These cytokines play a key role in immune responses, inflammation, and tissue repair, and thus serve as important indicators for assessing the anti-inflammatory effects of PA (Ignácio et al., 2019; Saxena et al., 2020). Microglia, as resident immune cells in the CNS, mediate inflammatory responses, maintain tissue homeostasis, and facilitate synaptic remodeling (Whitelaw et al., 2023). The complex interplay between microglia and cytokines is critical for maintaining CNS homeostasis and regulating neuroinflammation (Ferro et al., 2021). Therefore, during this period, exploring how PA modulates the pro-inflammatory functions of microglia to protect the brain from autoimmune neuroinflammation became a significant research focus (Zaychik et al., 2021). Additionally, the elderly population gradually emerged as a key focus in this field, as aging is a major risk factor for age-related diseases, including neurodegenerative disorders. Consequently, research on NDs also gained prominence. In the third phase, studies on NDs such as AD and PD proliferated. Recent findings have shown that PA can influence neuroinflammation and improve brain function by modulating the gut microbiota and its metabolites (Peng et al., 2024). PA also enhances neuroinflammation through multiple mechanisms, thereby creating more favorable conditions for the formation and maintenance of long-term potentiation (LTP), which in turn helps improve cognitive function and slow the progression of NDs (Lu et al., 2024).

Our findings on trend topics are consistent with those reported in previous studies. NDs are a heterogeneous group of complex disorders characterized by the loss of neurons and progressive degeneration in different regions of the nervous system (Cheng et al., 2021). NDs represent a significant global health issue, with an increasing incidence rate. Although the exact pathogenesis of NDs remains unclear, complex interactions between genetic, epigenetic, and environmental factors have been proposed (Agnello and Ciaccio, 2022). Understanding the molecular mechanisms underlying the pathogenesis of NDs is crucial for the development of effective therapeutic strategies. Some NDs are relatively common, such as AD and PD, while others, including Huntington’s disease (HD), amyotrophic lateral sclerosis (ALS), frontotemporal dementia (FTD), corticobasal syndrome (CBS), multiple system atrophy (MSA), and progressive supranuclear palsy (PSP), are rarer (Temple, 2023). Chronic neuroinflammation may be an underlying factor in many NDs. Studies have shown that modulating the glycolytic metabolism of microglia can influence their function, potentially reversing the central inflammatory responses associated with NDs (Huang et al., 2024). However, the specific mechanisms of microglial glycolysis in NDs still require further investigation, especially the primary and secondary relationships between changes in microglial functional phenotypes and glycolytic pathways, as well as the key timing and molecular targets of these changes. Recent studies have revealed bidirectional communication between the gut and muscles, in which irisin and muscle-derived brain-derived neurotrophic factor (BDNF) may mediate the positive effects of PA on certain aspects of AD pathophysiology through interactions with the gut microbiome ecosystem, neuroplasticity, anti-inflammatory signaling pathways, and neurogenesis (Cutuli et al., 2023; Sanchez-Martinez et al., 2024). Additionally, it has been found that exercise can improve cognitive deficits in ovalbumin (OVA)-sensitized rats, increase BDNF levels, and enhance LTP in the hippocampus of OVA-sensitized rats. LTP, a long-lasting enhancement of synaptic efficacy dependent on activity, has been implicated in NDs and is widely used to study synaptic plasticity (Wang et al., 2023; Abraham et al., 2024). However, the exact molecular mechanisms of synaptic plasticity have not yet been fully elucidated.

This finding regarding the progress of the theme of PA and NI is consistent with the existing literature. Traditionally, research has focused on how PA modulates the formation of neural connections to enhance cognitive function (Festa et al., 2023). However, recent research emphasis has shifted toward how PA regulates inflammation and immune responses within the CNS (Strohm and Majewska, 2024). On the other hand, the analysis of thematic evolution reflects the research boom in the field of PA and NI. In the lower-left quadrant, the theme of “Mental health” may be emerging. Regular physical exercise, cognitive engagement, a balanced diet, and social interaction are the key pillars for maintaining mental health (Dziewa et al., 2024). Exercise has beneficial effects on mental health and cognitive function, but the exact underlying mechanisms remain largely unknown.

The primary research themes identified in recent studies are “central-nervous-system,” “signaling pathway,” “microglial activation,” and “animal-model.” By establishing various animal models of CNS disorders, researchers have explored how PA modulates the activation of microglia and alters corresponding signaling pathways to ameliorate neuroinflammation. Chronic neuroinflammation, characterized by the persistent activation of microglia and the overproduction of pro-inflammatory cytokines, is a core pathological feature of many CNS diseases, such as AD, PD, and traumatic brain injury (TBI) (Stephenson et al., 2018). Microglia, the principal immune cells of the CNS, exhibit high plasticity and heterogeneity. Under normal physiological conditions, microglia remain in a “resting” state, monitoring changes in the CNS microenvironment through their highly branched morphology (Ginhoux et al., 2010). Upon injury or inflammatory stimulation to the CNS, microglia rapidly become activated, transitioning to an “activated” state, and release both pro-inflammatory cytokines (e.g., TNF-α and IL-1β) and anti-inflammatory cytokines (e.g., IL-10) to participate in the regulation of neuroinflammation (Pan et al., 2022). However, persistent activation may lead to chronic neuroinflammation, causing further neuronal damage. In recent years, the mechanisms by which physical exercise modulates the polarization state of microglia and the activity of signaling pathways to improve neuroinflammation have been validated through various animal models of CNS diseases.

In AD research, the APP/PS1 transgenic mouse model has demonstrated that PA intervention upregulates the BDNF/tropomyosin receptor kinase B (TrkB) signaling pathway and inhibits the activation of nuclear factor-κB (NF-κB). This results in reduced release of pro-inflammatory factors (such as TNF-α and IL-6) around β-amyloid (Aβ) plaques and increased expression of the anti-inflammatory factor IL-10 (Xu et al., 2022). Further studies have shown that PA regulates microglial metabolic reprogramming by activating the PI3K-AKT-mTOR signaling axis, thereby inhibiting their polarization toward the pro-inflammatory phenotype (M1) (Gao et al., 2023). In PD models, MPTP-induced PD mice subjected to treadmill training exhibit specific activation of the adenosine A2A receptor/cAMP-dependent protein kinase (PKA) signaling pathway. This significantly inhibits the assembly of the NLRP3 inflammasome and downstream IL-1β secretion, while promoting a metabolic shift in microglia from glycolysis to oxidative phosphorylation via AMPK-mediated metabolic conversion, thereby alleviating dopaminergic neuronal damage in the substantia nigra (He et al., 2024; Ishaq et al., 2024). MS research has elucidated the mechanism by which PA modulates microglial function through the gut microbiota–brain axis. In the experimental autoimmune encephalomyelitis (EAE) model, PA intervention increases short-chain fatty acids derived from gut microbiota (such as butyrate), inhibits STAT3 phosphorylation, and promotes the transformation of microglia toward an anti-inflammatory phenotype (M2), thereby alleviating demyelinating lesions (Parodi and Kerlero de Rosbo, 2021; Nakhal et al., 2024). In ischemic stroke models, early exercise training in transient middle cerebral artery occlusion (tMCAO) mice enhances the phagocytic function of microglia by activating the CX3CR1 signaling pathway, clearing apoptotic neuronal debris, and inhibiting acute inflammatory responses mediated by the HMGB1/TLR4 pathway (Zhu et al., 2022). Traumatic brain injury (TBI) research has further confirmed that controlled cortical impact (CCI) model mice subjected to PA intervention exhibit significantly reduced activity of the NF-κB signaling pathway, decreased release of pro-inflammatory factors, and accelerated recovery of neurological function (Singaravelu Jaganathan and Sullivan, 2022).

Despite the current research revealing the neuroprotective potential of PA, future studies need to further clarify the dose-response relationship between exercise intensity, frequency, and timing of intervention. Additionally, exploring the specific effects of different exercise modalities, such as resistance training and interval training, on microglial polarization is essential. Moreover, integrating single-cell sequencing with spatial transcriptomics to elucidate the heterogeneity of microglia, as well as conducting translational clinical studies to validate the conclusions from animal models, will provide a theoretical basis for precise exercise interventions in neuroinflammatory diseases.

## 5 Strengths and limitations

In this study, we employed bibliometric tools such as the Bibliometrix R package and VOSviewer to conduct an in-depth analysis of the countries, institutions, journals and keywords in the field of PA and neuroinflammation. We focused particularly on the trends, strategic distribution, and evolution of keywords, exploring the dynamic evolution of this field from multiple perspectives and clearly demonstrating the developmental trajectory and changes in the knowledge structure.

However, due to the limitations of the bibliometric tools used, the literature data in this study were sourced exclusively from the WoSCC database, which may limit the comprehensiveness of the findings. We acknowledge that relying solely on the WoSCC database may have certain limitations, as some relevant studies not indexed by this database were not included in our analysis. Nevertheless, the WoSCC database is widely recognized for its extensive coverage and high-quality standards in academic publishing. As recent similar bibliometric studies have shown, WoSCC remains a reliable source for bibliometric analysis in neuroscience research (Wang et al., 2024). We believe that our analysis based on WoSCC data effectively presents the overall landscape and trends in this field. Another limitation is the exclusion of non-English publications, which may have led to the omission of some relevant studies, which may have led to the omission of some relevant articles published in other languages. It is worth noting that the R-bibliometrix software package does not offer the Relative Citation Ratio (RCR), Field-Weighted Citation Impact (FWCI), or trends in the h-index for document analysis, which may to some extent affect the assessment of the influence of the documents.

## 6 Conclusion

The research scale in the field of PA and NI has significantly expanded, with an annual publication growth rate of 15.05%. China and the United States are the main contributing countries; however, the United States holds an advantage in terms of total citation counts and academic influence. Initially, this field focused on the biological mechanisms of neuroinflammation and hippocampal function, but in recent years, it has gradually shifted toward current directions such as NDs, microglial regulation, and the gut–brain axis. Research hotspots are now developing toward interdisciplinary integration and in-depth mechanistic exploration, with significant progress made in areas such as metabolic reprogramming, signaling pathway regulation, and validation through animal models. However, the study was limited by the use of a single database (WoSCC) and the exclusion of non-English literature. Future research should integrate data from multiple sources and expand multilingual literature analysis to provide a more comprehensive view of the field. The findings of this study provide a systematic scientific basis for scholars to grasp the disciplinary trends, optimize research directions, and formulate intervention strategies.

## Footnotes

1
http://www.bibliometrix.org

2
https://www.VOSviewer.com
