# Supplementary material for: Physical activity and neuroinflammation: a bibliometric analysis of research progress and future perspectives
Source: Front Aging Neurosci. 2025 Aug 4;17:1602724. doi: 10.3389/fnagi.2025.1602724 (PMC12358369; doi:10.3389/fnagi.2025.1602724)
Supplement: Supplementary file 1 [file Table_1.docx]

Most Influential Articles and Most Influential Authors

1. Most influential articles

In this study, we analyzed the collected articles using local citation (LC) and total citation (TC) scores. The TC represents the total number of citations an article has received across all databases, whereas the LC indicates the number of citations from articles within the research dataset. Normalized citation addresses the issue that newer works may not have had sufficient time to accumulate citations compared to earlier works(van Eck and Waltman, 2010).

Tables S1 and S2 present the rankings of the top 10 articles in the examined dataset based on total citations (TC), local citations (LC), and normalized citation counts. The article "Role of the Prefrontal Cortex in Pain Processing" has the highest TC value among the top 10 most globally cited articles. The article "Voluntary Exercise Promotes Glymphatic Clearance of Amyloid Beta and Reduces the Activation of Astrocytes and Microglia in Aged Mice" exhibits the highest LC value. Additionally, the article "Chronic exercise ameliorates the neuroinflammation in mice carrying NSE/htau23" has the highest LC/TC ratio. Moreover, based on normalized LC values, the article "Physical Exercise Inhibits Inflammation and Microglial Activation" is identified as the most cited article. Among original articles, "McKee AC, 2013, Brain" has the highest LC value of 64, while among review articles, "Smith DH, 2013, Nature Reviews Neurology" has the highest LC value of 13. Table S3 lists the top 10 most cited references based on citation counts. Among these frequently cited articles, "Wrann, CD, 2013, Cell Metabolism," "Heneka MT, 2015, Lancet Neurology," and "Lourenco MV, 2019, Nature Medicine" rank as the top three, with citation counts of 50, 49, and 43, respectively. Notably, among original research articles, "Wrann, CD, 2013, Cell Metabolism," "Lourenco MV, 2019, Nature Medicine," and "Choi SH, 2018, Science" are the top three most cited references. Among review articles, "Heneka MT, 2015, Lancet Neurology," "Erickson KI, 2011, Proceedings of the National Academy of Sciences of the United States of America," and "Cotman CW, 2007, Trends in Neurosciences" are the top three most cited references.

TABLE S1 Top 10 documents cited globally.

| **No.** | **Document** | **DOI** | **TC** | **TC per Year** | **Normalized TC** |
| --- | --- | --- | --- | --- | --- |
| 1 | ONG WY, 2019, MOL NEUROBIOL | 10.1007/s12035-018-1130-9 | 438 | 62.57 | 8.41 |
| 2 | BETTIO LEB, 2017, NEUROSCI BIOBEHAV R | 10.1016/j.neubiorev.2017.04.030 | 397 | 44.11 | 4.68 |
| 3 | NEWCOMBE EA, 2018, J NEUROINFLAMM | 10.1186/s12974-018-1313-3 | 371 | 46.38 | 7.16 |
| 4 | HORI H, 2019, PSYCHIAT CLIN NEUROS | 10.1111/pcn.12820 | 229 | 32.71 | 4.40 |
| 5 | HE XF, 2017, FRONT MOL NEUROSCI | 10.3389/fnmol.2017.00144 | 229 | 25.44 | 2.70 |
| 6 | ERION JR, 2014, J NEUROSCI | 10.1523/JNEUROSCI.4200-13.2014 | 229 | 19.08 | 2.57 |
| 7 | LI DJ, 2017, METABOLISM | 10.1016/j.metabol.2016.12.003 | 219 | 24.33 | 2.58 |
| 8 | XU YJ, 2016, BRAIN BEHAV IMMUN | 10.1016/j.bbi.2016.02.022 | 215 | 21.50 | 3.20 |
| 9 | AGUDELO LZ, 2018, CELL METAB | 10.1016/j.cmet.2018.01.004 | 208 | 26.00 | 4.01 |
| 10 | DE MIGUEL Z, 2021, NATURE | 10.1038/s41586-021-04183-x | 206 | 41.20 | 6.46 |

TC, total citations.

TABLE S2 Top 10 documents cited locally.

| **No.** | **Document** | **DOI** | **LC** | **TC** | **LC/TC** | **Normalized LC** |
| --- | --- | --- | --- | --- | --- | --- |
| 1 | HE XF, 2017, FRONT MOL NEUROSCI | 10.3389/fnmol.2017.00144 | 29 | 229 | 12.66 | 5.69 |
| 2 | KOHMAN RA, 2013, J NEUROINFLAMM | 10.1186/1742-2094-10-114 | 28 | 101 | 27.72 | 4.67 |
| 3 | MEE-INTA O, 2019, CELLS-BASEL | 10.3390/cells8070691 | 27 | 122 | 22.13 | 9.26 |
| 4 | BARRIENTOS RM, 2011, J NEUROSCI | 10.1523/JNEUROSCI.2266-11.2011 | 25 | 107 | 23.36 | 1.72 |
| 5 | KOHMAN RA, 2012, BRAIN BEHAV IMMUN | 10.1016/j.bbi.2011.10.006 | 25 | 129 | 19.38 | 3.29 |
| 6 | SPIELMAN LJ, 2016, BRAIN RES BULL | 10.1016/j.brainresbull.2016.03.012 | 25 | 91 | 27.47 | 5.15 |
| 7 | LEEM YH, 2011, BIOCHEM BIOPH RES CO | 10.1016/j.bbrc.2011.02.046 | 24 | 68 | 35.29 | 1.66 |
| 8 | LIU Y, 2020, J NEUROINFLAMM | 10.1186/s12974-019-1653-7 | 23 | 86 | 26.74 | 12.03 |
| 9 | JENSEN CS, 2019, EXP GERONTOL | 10.1016/j.exger.2019.04.003 | 20 | 77 | 25.97 | 6.86 |
| 10 | ZHANG XL, 2019, FRONT AGING NEUROSCI | 10.3389/fnagi.2019.00078 | 20 | 65 | 30.77 | 6.86 |

LC, local citations; TC, total citations.

TABLE S3 Top 10 most local cited references.

| **No.** | **References** | **DOI** | **LC** |
| --- | --- | --- | --- |
| 1 | WRANN CD, 2013, CELL METAB | 10.1016/J.CMET.2013.09.008 | 50 |
| 2 | HENEKA MT, 2015, LANCET NEUROL | 10.1016/S1474-4422(15)70016-5 | 49 |
| 3 | LOURENCO MV, 2019, NAT MED | 10.1038/S41591-018-0275-4 | 43 |
| 4 | ERICKSON KI, 2011, P NATL ACAD SCI USA, V108, P3017, DOI | 10.1073/PNAS.1015950108 | 42 |
| 5 | COTMAN CW, 2007, TRENDS NEUROSCI | 10.1016/J.TINS.2007.06.011 | 41 |
| 6 | CHOI SH, 2018, SCIENCE | 10.1126/SCIENCE.AAN8821 | 39 |
| 7 | GLEESON M, 2011, NAT REV IMMUNOL | 10.1038/NRI3041 | 39 |
| 8 | NICHOL KE, 2008, J NEUROINFLAMM | 10.1186/1742-2094-5-13 | 37 |
| 9 | DE LA ROSA A, 2020, J SPORT HEALTH SCI | 10.1016/J.JSHS.2020.01.004 | 34 |
| 10 | BOSTRÖM P, 2012, NATURE | 10.1038/NATURE10777 | 32 |

TC, total citations.

2. Most influential authors

At the author level, our analysis revealed the relationship between publication metrics and academic impact (Table S4). The h-index and total citations are two important indicators of academic impact, while the number of publications reflects an author's academic productivity. The top-ranked author is LI Y, who has the highest h-index of 8, with a total of 254 citations and 10 publications. GIL-MOHAPEL J and LEE Y are tied for second place, both with an h-index of 5. However, GIL-MOHAPEL J has a significantly higher total citation count (481) compared to LEE Y (209), despite both having published 5 papers.

TABLE S4 Top 10 most influential authors.

| **Rank** | **Author** | **h_index** | **TC** | **NP** |
| --- | --- | --- | --- | --- |
| 1 | LI Y | 8 | 254 | 10 |
| 2 | GIL-MOHAPEL J | 5 | 481 | 5 |
| 3 | LEE Y | 5 | 209 | 5 |
| 4 | LIU Y | 5 | 287 | 7 |
| 5 | MAIER SF | 5 | 413 | 5 |
| 6 | MEEUSEN R | 5 | 329 | 5 |
| 7 | WATKINS LR | 5 | 413 | 5 |
| 8 | WU CY | 5 | 222 | 5 |
| 9 | ARIDA RM | 4 | 135 | 5 |
| 10 | BARRIENTOS RM | 4 | 326 | 4 |

TC, total citations; NP, number of publications.

References

van Eck, N.J., and Waltman, L. (2010). Software survey: VOSviewer, a computer program for bibliometric mapping. *Scientometrics* 84(2)**,** 523-538. doi: 10.1007/s11192-009-0146-3.
